# Examining the Ability of Aerobic Halophilic Heterotrophic Microbial Consortia to Replace Ca by Mg in Different CaCO_3_ Precursors

**DOI:** 10.3389/fmicb.2022.791286

**Published:** 2022-03-21

**Authors:** Ammar Alibrahim, Maria Dittrich

**Affiliations:** Department of Physical and Environmental Sciences, University of Toronto Scarborough, Toronto, ON, Canada

**Keywords:** dolomite, sabkha, microbial diagenesis, enrichment experiment, halophile

## Abstract

Recent laboratory experiments have exhibited microbes as promising agents in solving the perplexing origin of ancient dolomite by demonstrating microbial capability to mediate dolomite nucleation and growth. However, dolomite crystals from laboratory experiments have shown irrelevant characteristics to ancient dolomite from mineralogical and petrological perspectives. A major irrelevant characteristic is that ancient dolomite was assumed to be formed after the replacement of Ca by Mg in precursor CaCO_3_ in a process known as diagenesis, which contrasts with the primary precipitation process observed in laboratory culturing experiments. Considering dolomite microbial experiments, one can imply the involvement of microbes in the formation of ancient dolomite, as microbes have shown the ability to overcome the dolomite kinetic barrier. Despite that fact, the ability of microbes in mediating dolomite diagenesis has not been investigated. In this study, microbes were applied to mediate replacement of Ca by Mg in different CaCO_3_ precursors. The microbial replacement experiments were based on the enrichment of aerobic halophilic heterotrophic microbial consortia sampled from sediments collected from Al-Subiya sabkha in Kuwait. Two experiments were performed in saturated media at 35°C for 14 and 30 days simulating the conditions of microbial dolomite experiments. The change in mineralogy was examined *via* powder X-ray diffraction (XRD), and the change in texture and compositional microstructures was examined using scanning electron microscopy with energy-dispersive spectroscopy (SEM-EDS). The effect of microbes on the alteration of CaCO_3_ precursors was studied by comparing biotic experimentations with abiotic controls. The biotic samples were shown to result in the favorable conditions for dolomite formation including an increase in pH and alkalinity, but no changes were observed in mineralogy or compositional microstructure of CaCO_3_ precursors. Our results suggest the inability of aerobic halophilic heterotrophic microbial consortia to introduce Mg replacement on CaCO_3_ precursors in a timely manner that is comparable to primary precipitation in microbial dolomite experiments. The inability of the enriched microbial consortia to mediate replacement can be ascribed to different factors controlling the diagenetic process compared to primary precipitation in microbial dolomite experiments.

## Introduction

Several laboratory studies have proposed the organogenic dolomite formation model after the successful precipitation of dolomite-like microscopic crystals by different microbial groups (Vasconcelos et al., 1995; Warthmann et al., 2000; Sánchez-Román et al., 2008; Kenward et al., 2013). The mechanism of microbial dolomite formation involves changing the chemistry of microbial surroundings (Dupraz et al., 2009) and providing nucleation sites for mineral growth either on microbial cell surfaces or the produced extracellular polymeric substances (EPS) (McCormack et al., 2018). The main motif of the organogenic model is the microbial ability to overcome the kinetic barrier of dolomite precipitation at low temperature simulating Earth’s surface conditions, where natural dolomite was commonly found in geologic settings (Vasconcelos and McKenzie, 1997).

Although microbial experiments have shown potential contributions toward solving the origin of dolomite in ancient sediments, different characteristics among laboratory dolomite and dolomite in nature require further explanation to conclude a unifying microbial mechanism for their involvement. First, precipitates of microbial experiments exhibit different morphologies of spheroids and dumbbell-shaped crystals (Vasconcelos et al., 1995; Warthmann et al., 2000; Kenward et al., 2013) in contrast to rhombic crystals of dolomite found in nature (Fairchild, 1983; Rosen and Coshell, 1992; Tucker, 1992). Second, the mode of dolomite formation in microbial experiments is direct primary precipitation from saturated solutions, which forms crystals in μm size; however, natural dolomite is a product of diagenetic replacement of precrusor calcium carbonate (Kaczmarek et al., 2017), which forms massive dolomite with a thickness that reaches >100 m (Ning et al., 2020). Therefore, it is likely that the crystal shape, mode of formation, and size are interrelated.

Most dolomites in nature are formed through chemical modifications of precursor carbonate rocks, limestone, or calcareous muds in a process called dolomitization expressed by the chemical equation: 2CaCO_3_ + Mg^2+^ ⇔ CaMg(CO_3_)_2_ + Ca^2+^ (Kaczmarek et al., 2017). Dolomitization is a process of carbonate diagenesis that occurs under near surface and shallow burial conditions (Jonas et al., 2017). In terms of hypersaline sabkha environments, the previaling sabkha model has proposed dolomitization as the replacement of precursor aragonite muds (Wells, 1962; Patterson and Kinsman, 1982). Nonetheless, the replacement of aragonite muds in the sabkha was only inferred from mass balance calculations (McKenzie, 1981), making sabkha diagenesis an equivocal assumption for dolomite formation (Hardie, 1987). Although modern dolomite formation was often linked to microbial involvement (Warthmann et al., 2000; Roberts et al., 2004; Sánchez-Román et al., 2008), the microbial role in dolomitizing pre-existing CaCO_3_ has not been investigated.

This work attempts to microbiologically replace Ca by Mg in different CaCO_3_ precursors at a low Earth’s surface temperature to mimic the mode of formation of ancient diagenetic dolomite. Microbes were proposed to be the “holy grail” catalyst of dolomitization in a laboratory room temperature experiment (Land, 1998), but their role in diagenesis was not proven yet. In this study, halophilic microbes were utilized as a mediator for the replacement reaction for the following reasons: (1) halophilic microbes thrive in fluids with high concentrations of Ca^2+^ and Mg^2+^ ions and increase the dolomite saturation index by producing HCO_3_^–^ (Sánchez-Román et al., 2008), and (2) halophilic microbes can provide free-carboxyl groups available for Mg^2+^ loading into carbonate crystals after dehydration of Mg[H_2_O]_6_^2+^ (Qiu et al., 2017). The halophilic microbial consortia in this work were enriched from Al-Subiya sabkha sediment where dolomite was found (Alkuwairan, 2013). Two experiments were carried out and three different calcium carbonate materials were tested. Change in mineralogy and compositional microstructure were examined using powder X-ray diffraction (XRD) and scanning electron microscopy with energy-dispersive spectroscopy (SEM-EDS), respectively. The trend of pH, alkalinity, and conductivity change through the incubation period was charted.

## Sampling Site and Sample Collection

**FIGURE 1:**
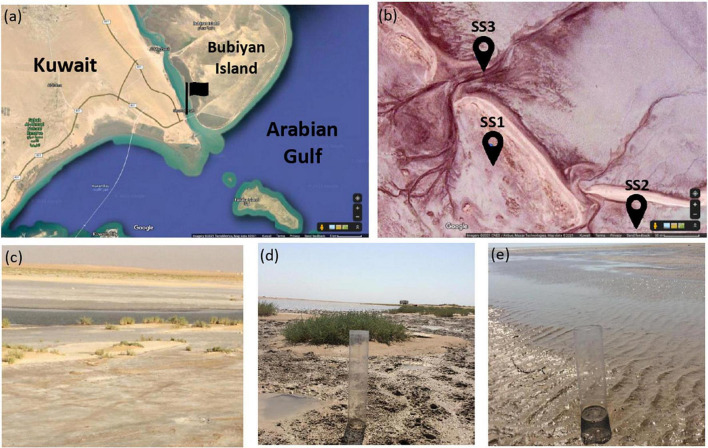
Location of the sampling site: **(a)** Kuwait map obtained from Google Maps and the flag points to Al-Subiya sabkha in north Kuwait. **(b)** Aerial image obtained from Google Maps of the three sampling zones indicated by the pins and the tidal channels in the sabkha are shown. **(c–d)** are representative field images of the sampling zones labeled as SSI, SS2, and SS3, respectively.

Al-Subiya area is located at the northeastern part of Kuwait between latitudes 29°22′ and 29° 40′ N and longitudes 47° 46′ and 48° 05′ E. This area is characterized by gravel plain, shallow depressions, sand plains, slopes, and coastal sabkha (El-Wahab and Al-Rashed, 2010). The coastal sabkha of Al-Subiya was described as a hypersaline environment harboring dolomite (Alkuwairan, 2013). Sediment cores and shallow seawater were sampled from three sites of the sabkha and were labeled as SS1, SS2, and SS3 for our experiment (Figure 1). Sediment cores were sampled using acrylic cores in which halophilic microbial consortia were enriched from the top 2 cm of the sediment layers. Seawater was analyzed to calculate dolomite saturation index in the sabkha and to prepare enrichment media simulating seawater characteristics of the sabkha. The temperature of seawater was measured *in situ* using a laboratory thermometer. Details on seawater analysis and results are in Supplementary Material 1.

## Methodology

**FIGURE 2:**
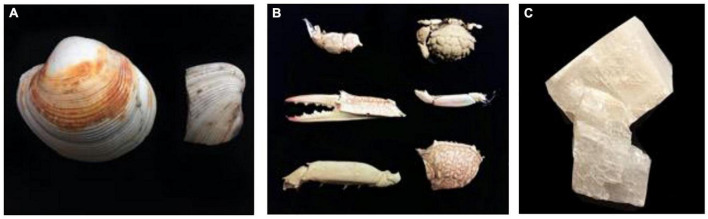
Starting materials used for the experiments: **(A)** bivalve shells, **(B)** crab skeleton parts, and **(C)** single-phase calcite.

**FIGURE 3:**
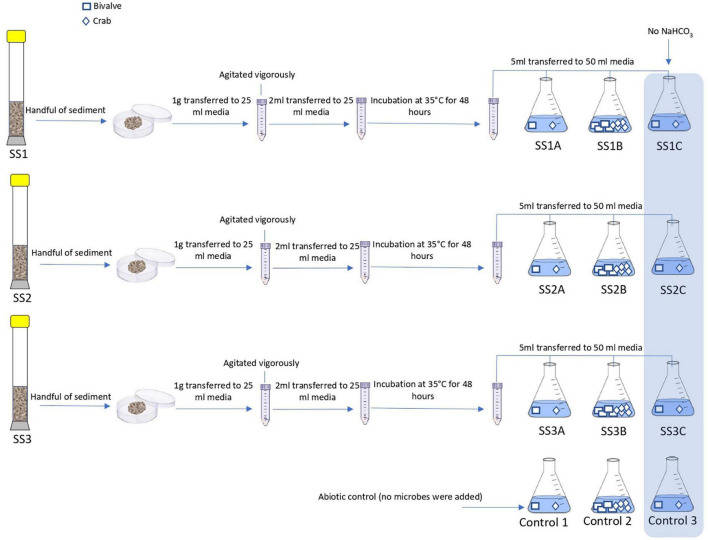
Schematic procedure of experiment A. The flasks SSI A, SS1B, and SS1C are 3 replicas of enriched sediment from core 1. The flasks SS2A, SS2B, and SS2B are 3 replicas of enriched sediment from core 2. The flasks SS3A, SS3B, and SS3C are 3 replicas of enriched sediment from core 3. Control 1, Control 2, and Control 3 are the abiotic samples.

Microbes from the sediment were enriched in nutritious media to support their growth and activity. The composition of the media was prepared to selectively grow halophilic heterotrophic microbial consortia. Details about the process of microbial enrichment are in Supplementary Material 2. Different CaCO_3_ precursors of bivalve shell, crab skeletons, and single-phase calcite were used in this experiment to examine microbial ability to mediate replacement (Figure 2). Further details on the preparation of starting materials are in Supplementary Material 3. Experiment (A) comprised nine experimental biotic samples and three control abiotic samples. The experimenting time was for 14 days where microbes were enriched from three cores. The tested starting materials were bivalve shell and crab skeletons (Figure 3). The pH of the media was measured before and after the experiment (Table 1). Mineralogy, textures, and chemical microanalysis of starting martials were examined before and after the experiment. More details on sample labeling and experiment design are in Supplementary Material 4.1. Experiment (B) comprised four biotic samples, four abiotic controls with organics (media), and four abiotic saline control (Supplementary Figure 1). The experiment was running for 30 days. pH, alkalinity, and conductivity were measured throughout the experimenting time. Texture and compositional microanalyses were analyzed after incubation. More details on sample labeling and experiment design are in Supplementary Material 4.2. Powder X-ray diffraction (XRD) was conducted to analyze change in CaCO_3_ mineralogy, while scanning electron microscopy with energy-dispersive spectroscopy (SEM-EDS) was used to detect change in texture and compositional structure. Details of sample preparation and machines settings are in Supplementary Material 5.

**TABLE 1:** pH values before running the experiment expressed as *t* = 0 and after finishing the experiment expressed as *t* = 14, as well as pH change (△) after 14 days of incubation in the experiment.

#	Sample ID	*t* = 0	*t* = 14	△
				
1	SS1A	7.2	8.77	1.57
2	SS1B	7.2	8.74	1.54
3	SS1C	7.2	8.77	1.57
4	SS2A	7.2	8.6	1.4
5	SS2B	7.2	8.65	1.45
6	SS2C	7.2	8.58	1.38
7	SS3A	7.2	8.71	1.51
8	SS3B	7.2	8.82	1.62
9	SS3C	7.2	8.85	1.65
10	Control A	7.2	7.23	0.03
11	Control B	7.2	7.57	0.37
12	Control C	7.2	7.13	−0.07

## Results

### Experiment A: 14 Days of Incubation

#### pH Change After Incubation

The pH considerably increased in all enriched samples with an average increase in 1.52 (σ = 0.09), but insignificant changes were observed in the control samples (Table 1).

#### Mineralogical, Texture, and Compositional Microstructure

**FIGURE 4:**
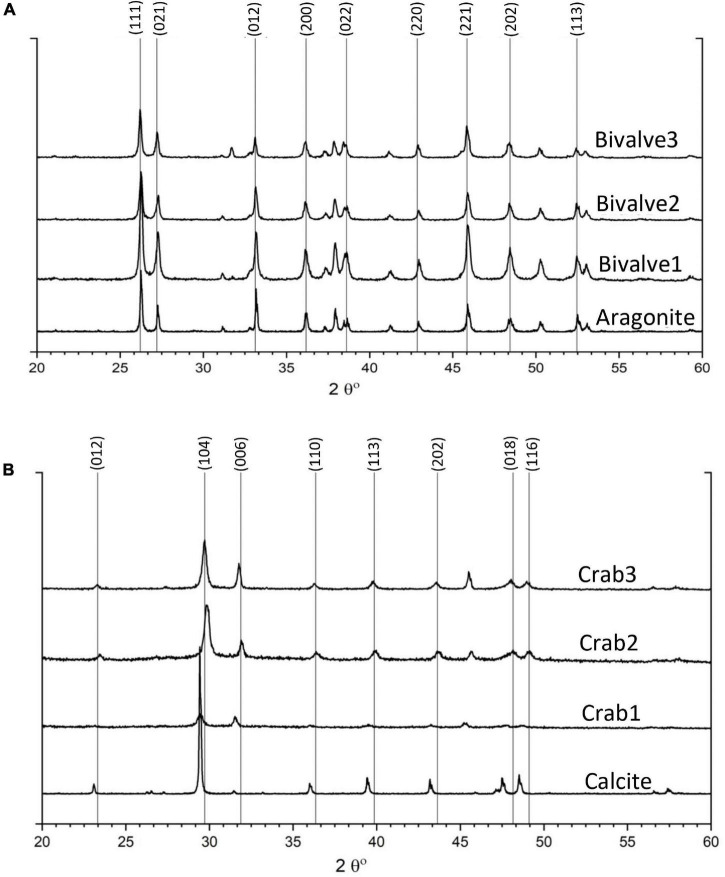
XRD results of starting materials prior to incubation: **(A)** different fragments of bivalve showing single-phase aragonite in comparison to RRUFF aragonite and **(B)** different parts of crab skeleton showing Mg-calcite with minor but varying Mg concentration in comparison to standard calcite from RRUFF database.

X-ray diffraction for mineralogical analysis of bivalve shells and crab skeletons was performed before and after the experiment. Before the experiment, the results of three random bivalve fragments indicated single-phase aragonite in comparison to the ICDD and RRUFF aragonite before the experiment (Figure 4A). Additionally, three parts of the crab skeleton revealed calcite with minor but varying magnesium contents ranging from Ca^0.845^Mg^0.155^CO_3_ to Ca^0.94^Mg^0.006^CO_3_ based on ICDD matches and compared to the standard RRUFF calcite (Figure 4B).

**FIGURE 5:**
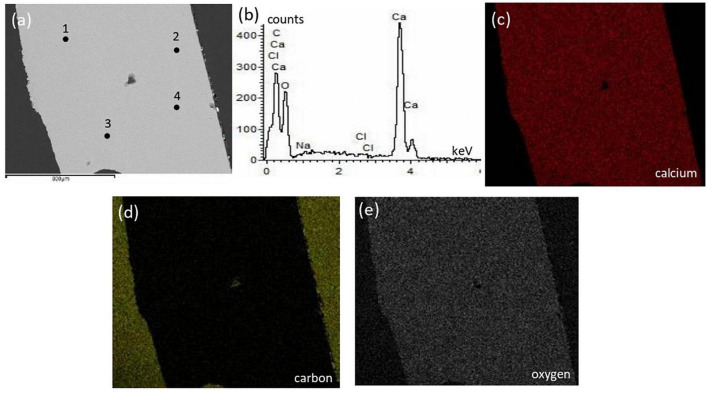
Textural characterization and microchemical analysis of a representative bivalve sample: **(a)** electron image showing homogeneous texture with no cavities; **(b)** representative EDS spectra of the 4 points indicated in the electron image; **(c–e)** are EDS mapping of calcium, carbon, and oxygen, respectively.

**FIGURE 6:**
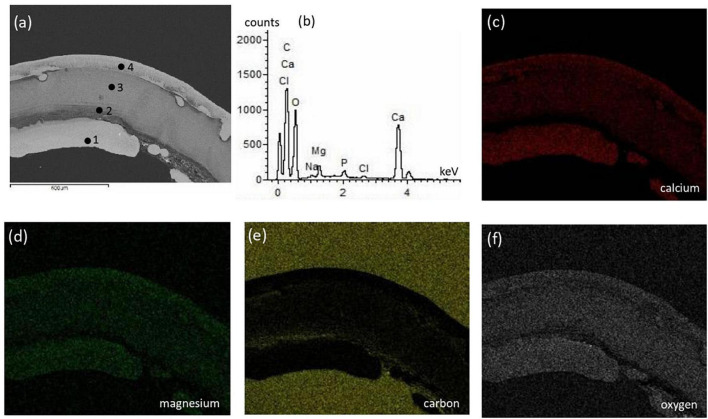
Textural characterization and microchemical analysis of a representative crab skeleton fragment: **(a)** electron image showing heterogenous color contrast related to varying elements abundance; **(b)** representative EDS spectra of the 4 points indicated in the electron image; **(c–f)** are EDS mapping of calcium, magnesium, carbon, and oxygen, respectively. The different parts of crab shell have varying elemental composition where the spectra in **(b)** represents the main elements.

The surficial texture of a representative bivalve shell was fairly smooth and homogeneous with no porosity or cavities, and the compositional microstructures displayed peaks of calcium, oxygen, and carbon with an absence of magnesium (Figure 5). Conversely, the crab skeleton displayed porous and heterogeneous textures and was composed of calcium, carbon, oxygen, and magnesium (Figure 6).

There was no mineralogical change in the bivalve samples after incubation, as it revealed the same single-phase aragonite prior to incubation (Supplementary Material 6). The crab skeleton after incubation displayed Mg-calcite skeleton with no perceived change in mineralogy (Supplementary Material 6). Peaks right shifting of the crab samples in comparison to calcite is due to minor magnesium content in the crab’s skeleton (Gregg et al., 2015). SEM-EDS of the representative bivalve sample showed minor heterogeneity (likely due to NaCl), no development of pores/cavities, and no magnesium integration on the bivalve surface (Supplementary Material 7). Any change of the crab skeleton texture was considered inconclusive due to the presence of pre-existing magnesium, since it is a constituent of crab shells (S7). As EDS is a semi-quantitative analysis, we were unable to determine the change in Mg content in crab skeletons.

### Experiment B: 30 Days of Incubation

#### Dynamics of pH, Alkalinity, and Conductivity During the Experiments

**FIGURE 7:**
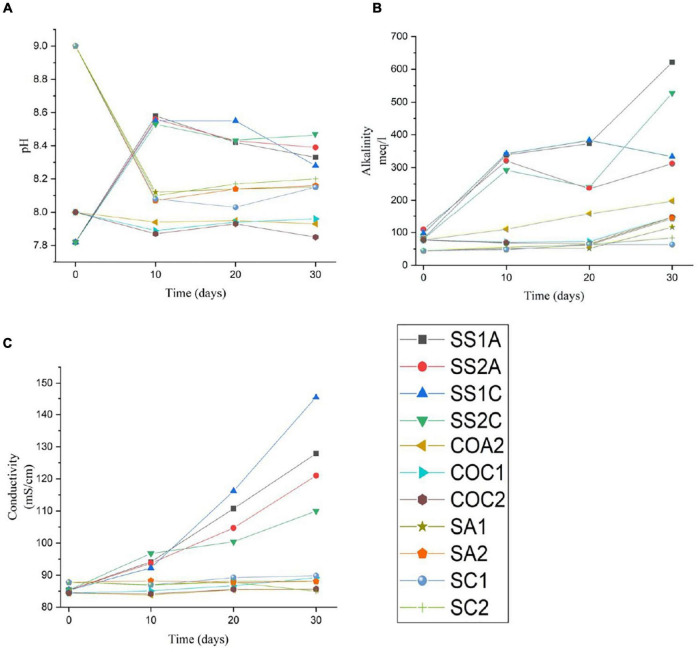
Charted change in pH **(A)**, change in alkalinity **(B)**, and change in conductivity **(C)** in 11 samples throughout the incubation period of 30 days. The enriched samples are SSA1, SS2A, SS1C, and SS2C. The abiotic organic controls are COA2, COC1, and COC2. The abiotic saline controls are SA1, SA2, SCI, and SC2.

A significant increase in pH, alkalinity, and conductivity was observed in all enriched samples (SS1A, SS2A, SS1C, and SS2C). After day 10, the control media COA1 was turbid and accidentally contaminated due to multiple handling, which resulted in changing pH and alkalinity. The pH, alkalinity, and conductivity of the other control media COA2, COC1, and COC2 remained almost constant. The pH of the saline control SA1, SA2, SC1, and SC2 dropped from 9 to an average pH of 8.17, the conductivity remained almost constant, and the alkalinity showed an increase after day 20 in all samples where alkalinity was higher in aragonite samples SA1 and SA2 compared to calcite samples SC1 and SC2. The results of pH, alkalinity, and conductivity throughout the incubation period are shown in Table 2 and Figure 7.

**TABLE 2:** pH, alkalinity, and conductivity change throughout incubation for 11 samples measured at *t* = 0, *t* = 10, *t* = 20, and *t* = 30 days.

#	Sample	pH				Alkalinity (meq/L)				Conductivity (mS/cm)			
		*t* = 0	*t* = 10	*t* = 20	*t* = 30	*t* = 0	*t* = 10	*t* = 20	*t* = 30	*t* = 0	*t* = 10	*t* = 20	*t* = 30
1	SS1A	7.82	8.58	8.42	8.33	80.58	338.38	372.80	620.96	85.33	94.14	110.7	127.9
2	SS2A	7.82	8.56	8.43	8.39	108.90	320.17	235.05	311.81	85.33	93.71	104.7	121
3	SS1C	7.82	8.55	8.55	8.28	96.68	342.06	382.69	333.04	85.33	92.24	116.2	145.5
4	SS2C	7.82	8.53	8.43	8.47	79.54	292.13	239.83	527.77	85.33	96.76	100.4	110
5	COA2	8	7.94	7.95	7.93	76.92	109.11	157.72	197.53	84.45	83.83	85.41	85.54
6	COC1	8	7.89	7.94	7.96	76.92	69.44	71.42	146.66	84.45	85.12	86.68	89.2
7	COC2	8	7.87	7.93	7.85	76.92	68.02	64.81	148.11	84.45	84.19	85.54	85.65
8	SA1	9	8.12	8.14	8.15	44.05	51.89	51.32	115.95	87.77	86.9	88.23	88.11
9	SA2	9	8.07	8.14	8.16	44.05	48.65	61.89	142.15	87.77	88.24	87.71	88.06
10	SC1	9	8.08	8.03	8.15	44.05	47.44	62.89	63.01	87.77	86.97	89.25	89.8
11	SC2	9	8.1	8.17	8.2	44.05	54.88	63.01	83.21	87.77	87	87.74	84.95

*The enriched samples are SSA1, SS2A, SS1C, and SS2C. The abiotic organic controls are COA2, COC1, and COC2. The abiotic saline controls are SA1, SA2, SC1, and SC2.*

#### Texture and Compositional Microstructure

**FIGURE 8:**
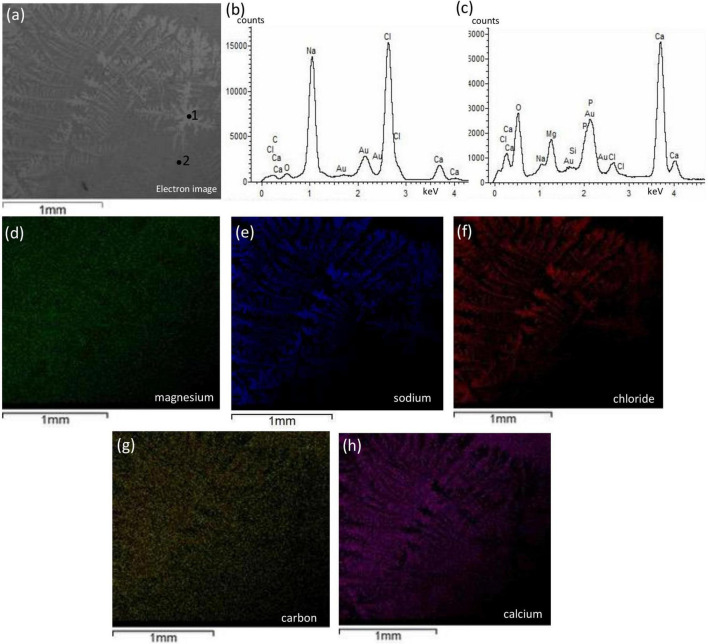
Textural characterization and microchemical analysis of bivalve with enriched halophilic consortia, SSA1 sample. The dendritic structure labeled with #1 showing in panel **(a)** is attributed to NaCl integration and inferred from EDS spectra in panel **(b)** while the background labeled with #2. The EDS mapping of magnesium in panel **(d)** shows non-preferential magnesium precipitation with no diagenetic features. The mapping of sodium and chlorine in panels **(e,f)**, respectively, are co-localized indicating NaCl precipitation with dendritic structure. Panels **(g,h)** represent the co-localized mapping of carbon and calcium, respectively, and show to be the integral for the dendritic structure.

Figure 8 shows representative SEM-EDS images of bivalve (more results are in Supplementary Material 7.2). We observed no change in texture, no development of cavities, and no change in compositional microstructures. However, some color contrast heterogeneity was observed in CaCO_3_ matrix (e.g., SSA1) and was related to NaCl integration (Figure 8). Supplementary Figures 7, 8 show surficial microcrystal precipitates, and no porosity/cavities were observed.

## Discussion

Our proposed microbial replacement experiment was set forth in an attempt to answer a long-standing geological question about the ancient origin of dolomite from a biological perspective. The replacement experimentation was based on the diagenetic formation mode of ancient dolomite where Mg substituted Ca in limestone rocks (Daly, 1907; Van Tuyl, 1916). Furthermore, the examination of microbial ability to mediate replacement at low temperature (<50°C) was inspired from laboratory experiments that have shown the successful formation of microbial dolomite or one of dolomite’s phases (Roberts et al., 2004; Sánchez-Román et al., 2009; Bontognali et al., 2010, 2014; Deng et al., 2010; Krause et al., 2012; Brauchli et al., 2016; Petrash et al., 2017; Alibrahim et al., 2019). Therefore, we found it reasonable to examine the ability of microbes to replace precursor calcium carbonates, as microbes have potentials toward overcoming the kinetic barrier of dolomite; such replacement experiments have not been previously conducted. Therefore, whether microbes are capable to mediate CaCO_3_ replacement to form dolomite or not is an open question.

The geological implication of this study can uphold the microbial diagenetic model of dolomite formation in Precambrian rocks of Isua supracrustal belt (W. Greenland) where the formation of low-temperature and pre-metamorphic dolomite demonstrates non-metasomatic features that are associated with diagenetic replacement (Nutman et al., 2010). The successful *in vitro* microbial Mg replacement of CaCO_3_ is a consequential evidence and a fingerprint of early life back to 3.7 Ga where microbes have mediated the diagenetic low-temperature dolomite in Precambrian Isua supracrustal belt (Nutman et al., 2010).

In experiments A and B, the enriched samples with microbes showed an increase in pH, which can be related to amino acid metabolism that produces NH_3_, creating an alkaline environment (Sánchez-Román et al., 2008). As for experiment B, the enriched samples showed an increase in conductivity compared to abiotic samples, which can be related to the activity of microbial multi-enzyme systems associated with peptone breaking down into smaller amino acids, which are consumed by microbes to support their growth. This increase in conductivity is in parallel to the concentration of amino acids (Krishnamurti and Kate, 1951).

In this study, the aerobic halophilic heterotrophic microbial consortia were unable to carry on Mg replacement to precursor calcium carbonates despite the oversaturated conditions with respect to dolomite. The incubation time for the experiments was designated for a timely fashion similar to successful microbial dolomite experiments (Table 3).

**TABLE 3:** Examples of successful laboratory microbial dolomite experiments showing the formation of dolomite or one of dolomite’s phases via different microorganisms within 30 days of incubation.

Microbial group	Microorganisms’ identity	Biomineral	Temperature	Incubation time	pH change	References
Moderately halophilic aerobic bacteria	*Halomonas meridiana Virgibacillus marismortui*	Dolomite	25/35°C	30 days	7.2 → 9	Sánchez-Román et al., 2008
Sulfate-reducing bacteria	*Desulfobulbus mediterraneus*	Mg-rich dolomite	21°C	14 days	7.4 → 7.8	Krause et al., 2012
Sulfate-reducing bacterium	*Desulfotomaculum ruminis*	Dolomite	37°C	1 month	7.8 → 9.4	Deng et al., 2010
Moderately halophilic aerobic bacteria	*Shewanella piezotolerans*	Disordered dolomite	25°C	10 days	7.6–7.8 → 9	Huang et al., 2019
Sulfate-reducing bacterium	*Desulfonatronovibrio hydrogenovorans*	Partially ordered dolomite	30°C	30 days	pH increased	Warthmann et al., 2000
Halophilic archaeon	*Haloferax volcanii*	Dolomite	45°C	80 h	ND	Qiu et al., 2017

Many reasons can stand behind this failure, individually and collectively. First, the replacement reactions require a pumping system of fluids with a high Mg content in order to be able to dolomitize calcium carbonates (Ning et al., 2020). In our experiments, we had a limited volume of fluids for replacement reactions (≥75 ml). Second, low temperature of dolomite-replacive reactions require long reaction times (>10^4^ years) due to kinetic factors (Hardie, 1987). The scarcity of dolomite in modern marine environments can further support the kinetic issue of dolomite (Land, 1998). Third, microbes failed to introduce cavities as reactive surfaces in the CaCO_3_ starting materials where the reactive surfaces are important for the permeation of the dolomitizing fluids. A hydrothermal dolomitization experiment demonstrated that the reaction rate of Ca replacement by Mg in carbonate is proportional to the development of porosity, cavities, and trench within the rims of reactive surfaces (Jonas et al., 2017). Fourth, the limited microbial biomass may have been unable to break the hydration shell of Mg^2+^–H_2_O complexes (Yang et al., 2012); therefore, the availability of Mg^2+^ ions for dolomitization was restricted. Fifth, it is important to note that the high-resolution chemical and morphological characteristics of starting materials is a crucial factor in dolomitization. For example, the finer is the grain size, the more susceptible is calcium carbonate to dolomitization (Van Tuyl, 1916). In our experiments, the different calcium carbonate precursors look impermeable, and fine grains were not observed. Lastly, as CaCO_3_ replacement by dolomite is a coupled dissolution-recrystallization process (Montes-Hernandez et al., 2014), the initial dissolution step was not possible due to saturation conditions of the media.

This work can be considered as a cornerstone for future microbial dolomitization studies that can investigate microbial ability to overcome dolomitization kinetics. Future experiments for microbial dolomitization at ambient Earth’s surface conditions can examine different CaCO_3_ starting materials such as karst, limestone, and coral skeleton. Furthermore, other microbial groups, such as cyanobacteria, can be used for future replacement experiments, as cyanobacteria can concentrate the level of magnesium preferentially on their sheath (Gebelein and Hoffman, 1973). Moreover, cyanobacteria can catalyze dolomite formation by their metabolic activity such as photosynthetic electron transport in the thylakoids, which results in pH increase within their intimate environment and consequently increasing dolomite saturation index (Kamennaya et al., 2012). We believe that microbial replacement of Ca by Mg in CaCO_3_ precursors is governed by different factors in comparison to the primary precipitation process. The importance of Mg abundance, longer reaction time, formation of reactive surfaces, high microbial biomass, fine-grain CaCO_3_ precursors, and episodic periods of dissolution and recrystallization reactions and their synergy toward low-temperature microbial replacement reactions require further investigations.

## Conclusion

Here, we performed experiments aimed to study the role of aerobic halophilic microbial consortia in replacement of Ca by Mg in calcium carbonate precursors at low temperature. The samples with microbial enrichment showed increased pH and alkalinity in a supersaturated media, and no replacement was observed neither *via* XRD nor SEM-EDS. The inability of the enriched microbial consortia to replace Ca by Mg in CaCO_3_ precursors can be related to the short timeline of our experiments. Indeed, secondary replacement is a lengthy process compared to dolomite primary precipitation experiments. The precursor CaCO_3_ materials were neither porous or permeable nor microbes were able to introduce porosity/permeability that would increase reactive surfaces for dolomitizing fluids to flow and conduct replacement. Finally, the controlling factors of microbial dolomite primary precipitation are likely dissimilar to the controlling factors of replacive microbial dolomite.

## Data Availability Statement

The original contributions presented in the study are included in the article/Supplementary Material, further inquiries can be directed to the corresponding author.

## Author Contributions

AA and MD contributed to the conception of the research and study design, involved in data interpretation, preparation of the final draft of the manuscript to be published, agreed to be accountable for all aspects of the work, and ensuring that questions related to the accuracy or integrity of any part of the work are investigated and resolved appropriately. AA was responsible for conducting all laboratory experiments, collecting data, data analysis, and writing the first draft of the manuscript. Both authors contributed to the article and approved the submitted version.

## Conflict of Interest

The authors declare that the research was conducted in the absence of any commercial or financial relationships that could be construed as a potential conflict of interest. The handling editor is currently organizing a research topic with one of the author MD.

## Publisher’s Note

All claims expressed in this article are solely those of the authors and do not necessarily represent those of their affiliated organizations, or those of the publisher, the editors and the reviewers. Any product that may be evaluated in this article, or claim that may be made by its manufacturer, is not guaranteed or endorsed by the publisher.
