# Supplementary material for: Examining the Ability of Aerobic Halophilic Heterotrophic Microbial Consortia to Replace Ca by Mg in Different CaCO3 Precursors
Source: Front Microbiol. 2022 Mar 21;13:791286. doi: 10.3389/fmicb.2022.791286 (PMC8977737; doi:10.3389/fmicb.2022.791286)
Supplement: Supplementary file 1 [file Data_Sheet_1.pdf]

Supplementary

S1. Seawater analysis

A volume of 4 ml of seawater was transferred to glass scintillation vials and mixed with 15 μl of 2.5% $HgCl_2$ for the analysis of total alkalinity using 905 Titrando, Metrohm and Tiamo 2.2 software (with acid concentration = 0.1 N HCl). Additional seawater was transferred to 50 ml tubes to measure pH, conductivity, cations, and anions. All seawater samples were preserved at 4 ºC and sent to the lab on the same day of sampling to conduct analyses. pH and conductivity were measured using SevenGo Duo SG23 Mettler Toledo GmbH, metals concentrations were measured using ICP-OES Horiba Jobin Yvon Type Ultima 2, and chloride, silica, and sulphate anions were analysed using HACH DR 6000 spectrophotometer. The samples were diluted 10-folds by mixing 5 ml of stock sample with 45 ml Milli-Q water before digestion and analysis. The final volume of 50 ml was digested with 0.25 ml HNO_3 + 2.5 ml HCl on a hot plate. After cooling down, the samples were analysed by ICP-OES using 3 points for the calibration curve. An exceptional dilution of 100-folds was used for magnesium and sodium as the concentration of the analytes were higher than the calibration range and silica and sulphate were diluted 125- and 10-folds, respectively. Dolomite saturation index was calculated using an aqueous geochemical modelling software (PHREEQC). Supplementary table 1 shows the results of the seawater analysis and dolomite saturation indices.

Supplementary table 1: Results of sabkha seawater analysis of SS1, SS2, and SS3 zones. Dolomite saturation index is calculated from PHREEQC software.

#	Parameter		Sample ID		
			SS1	SS2	SS3
1	pH		7.92	7.99	8.11
2	Alkalinity	meq/L	34.58	55.41	98.59
		mg/l ($CaCO_3$)	173.06	277.32	493.42
3	Conductivity (mS/cm)		71.7	75.5	86.4
4	Salinity (‰)		43.3	46.0	53.7
5	Temperature (ºC)		30.5	30.5	31
6	Calcium (mg/l)		643.7	731.0	767.3
7	Magnesium (mg/l)		1975.4	2151.3	2462
8	Sodium (mg/l)		17379	17439	19954.3
9	Potassium (mg/l)		632.0	684.7	795.0
10	Strontium (mg/l)		12.8	13.85	15.4
11	Boron (mg/l)		5.5	6.1	6.84
12	Sulphate (mg/l)		4573	5258	5813
13	Silicon (from silica) (mg/l)		1.24	1.60	1.58
14	Chloride (mg/l)		28782	30626	40816
15	Dolomite SI		2.66	3.26	4.05

S2. Microorganisms

Oxygen in the headspace of the horizontally shaking flasks provides microbes with the aerobic conditions. To prevent any external microbial contamination, the sampled core was opened in a biosafety cabinet under aseptic conditions and a handful of the sediment was taken from the middle of the core using a sterile spatula transferred to a Petri dish. One gram of the sediment was weighed, transferred to 25 mL media, and shaken vigorously for 1 minute to release the microbes attached to the sediment particles. Next, 2 ml of the supernatant was transferred to a new 25 ml media and incubated at 35 °C for 48 hours to allow the microbes to adapt to the media. Lastly, 5 mL from the incubated media was transferred to 50 ml media in experiment A (figure 3) and to 75 ml in experiment B (Supplementary figure 1) to start the experimentation.

S3. Starting Materials

Bivalve and crab skeletons were collected from Al-Subiya sabkha and assigned as biogenic $CaCO_3$ starting materials where single-phase calcite was provided by the Department of Earth Sciences, University of Toronto (Figure 2). The $CaCO_3$ materials were cut into 4 x 4 x 4 mm blocks and were sterilised using UV light for 1 hour per fragment side. Sterility of the starting materials was confirmed by the absence of microbial growth when 3 random blocks were incubated at 35 °C for 48 hours in sterile liquid media.

S4. Experiments designs

S4.1 Experiment A: 14 Days of Incubation

The enrichment media was modified from D1 media based on the highest salinity concentration of SS3 seawater. The composition of the media was: $MgSO_4.7H_2O$ (2.2 g), $MgCl_2$ anhydrous (8.8 g), $CaCl_2.2H_2O$ 9 (2.1 g), $NaHCO_3$ (0.11 g), NaCl (50 g), yeast extract (10 g), proteose peptone (5 g), glucose (1 g), and de-ionized water was added to complete 1 litre. After autoclaving, the media was adjusted to pH 7.2 by sterile 0.5 M NaOH. The Mg/Ca ratio was 4.3 and dolomite saturation index was 2.82. The experiment comprised a total of twelve 125 ml autoclaved Erlenmeyer flasks whereby all flasks contained 50 mL media. Using 125 ml Erlenmeyer flasks, experiment A comprised 9 experimental biotic samples and 3 control abiotic samples (Figure 3). Incubation time was designed for 14 days on a platform mechanical shaker at 35 ºC. Samples labelling and abbreviation are in supplementary table 2. Each flask of SS1A, SS1C, SS2A, SS2C, SS3A, SS3C, Control1 and Control3 contained 2 pieces of starting materials, one bivalve and one crab for SEM-EDS investigation. Each flask of SS1B, SS2B, SS3B, and Control2 contained 5 pieces of bivalve and 5 pieces of crab for XRD investigation (the collection of the 5 pieces of bivalve and the 5 pieces of crab, separately, were considered as one bivalve sample and one crab sample after pulverization for XRD). One set of flasks were examined with no $NaHCO_3$ in order to compare the microbial effect on pH, which was measured after the experiment.

Supplementary table 2: Identifying samples labelling and abbreviation of experiment A

Label	Referred to	Label	Referred to
SS1A	Enriched sediment surface core 1 replica 1	SS3A	Enriched sediment surface core 3 replica 1
SS1B	Enriched sediment surface core 1 replica 2	SS3B	Enriched sediment surface core 3 replica 2
SS1C	Enriched sediment surface core 1 replica 3	SS3C	Enriched sediment surface core 3 replica 3
SS2A	Enriched sediment surface core 2 replica 1	Control 1	Sterile media abiotic control replica 1
SS2B	Enriched sediment surface core 2 replica 2	Control 2	Sterile media abiotic control replica 2
SS2C	Enriched sediment surface core 2 replica 3	Control 3	Sterile media abiotic control replica 3

S4.2 Experiment B: 30 Days of Incubation

The enriched media from experiment A was modified to have a higher Mg/Ca ratio whilst maintaining the salinity of the SS3 site (60 ‰). The concentration of yeast extract and proteose peptone was reduced to one third to eliminate the possible effect of organics on biomineralization. The composition of the media was: $MgCl_2$ anhydrous (23.8 g), $CaCl_2.2H_2O$ (0.73 g), $NaHCO_3$ (0.46 g), NaCl (35 g), yeast extract (3.4 g), proteose peptone (1.7 g), glucose (1 g), and de-ionized water was added to complete 1 litre. The pH was adjusted with sterile 0.5 M NaOH to 7.82 in the enriched samples, 8.0 in the organic controls, and 9.0 in the saline controls. The Mg/Ca ratio = 23 and the dolomite saturation index = 3.09 in the enriched media and organic controls while the dolomite saturation index = 4.79 in the saline controls. The crab fragments were substituted for single-phase calcite and the microbes were enriched from two cores only. The incubation period was implemented for 30 days. One side of each fragment of bivalve and single-phase calcite was ground with 600 grit size sandpaper then polished with 1200 grit size sandpaper. Samples labelling and abbreviation are in supplementary table 3. After the incubation period, the polished side of each fragment was labelled using engraving steel pen for SEM-EDS investigations. Each Erlenmeyer flask contained 75 m of the media. The pH, alkalinity, and conductivity were measured through the incubation period at t = 0, t = 10, t = 20, and t = 30 days. The experiment comprised 4 biotic experimenting samples SSA1, SS2A, SS1C, and SS2C. Two types of control were used: 1) abiotic control samples (sterilized media) of COA1, COA2, CO1, and COC2, and 2) abiotic sterile saline SA1, SA2, SC1, and SC2. The varying starting pH values were set in order to monitor microbial limit of controlling pH change.

Supplementary table 3: Identifying samples labelling and abbreviation of experiment B

Label	Referred to	Label	Referred to
SS1A	Enriched sediment surface1 bivalve	COC1	Control media with organics calcite1
SS2A	Enriched sediment surface2 bivalve	COC2	Control media with organics calcite2
SS1C	Enriched sediment surface1 bivalve	SA1	Saline media bivalve 1
SS2C	Enriched sediment surface2 calcite	SA2	Saline media bivalve 2
COA1	Control media with organics bivalve	SC1	Saline media calcite 1
COA2	Control media with organics bivalve	SC2	Saline media calcite 2

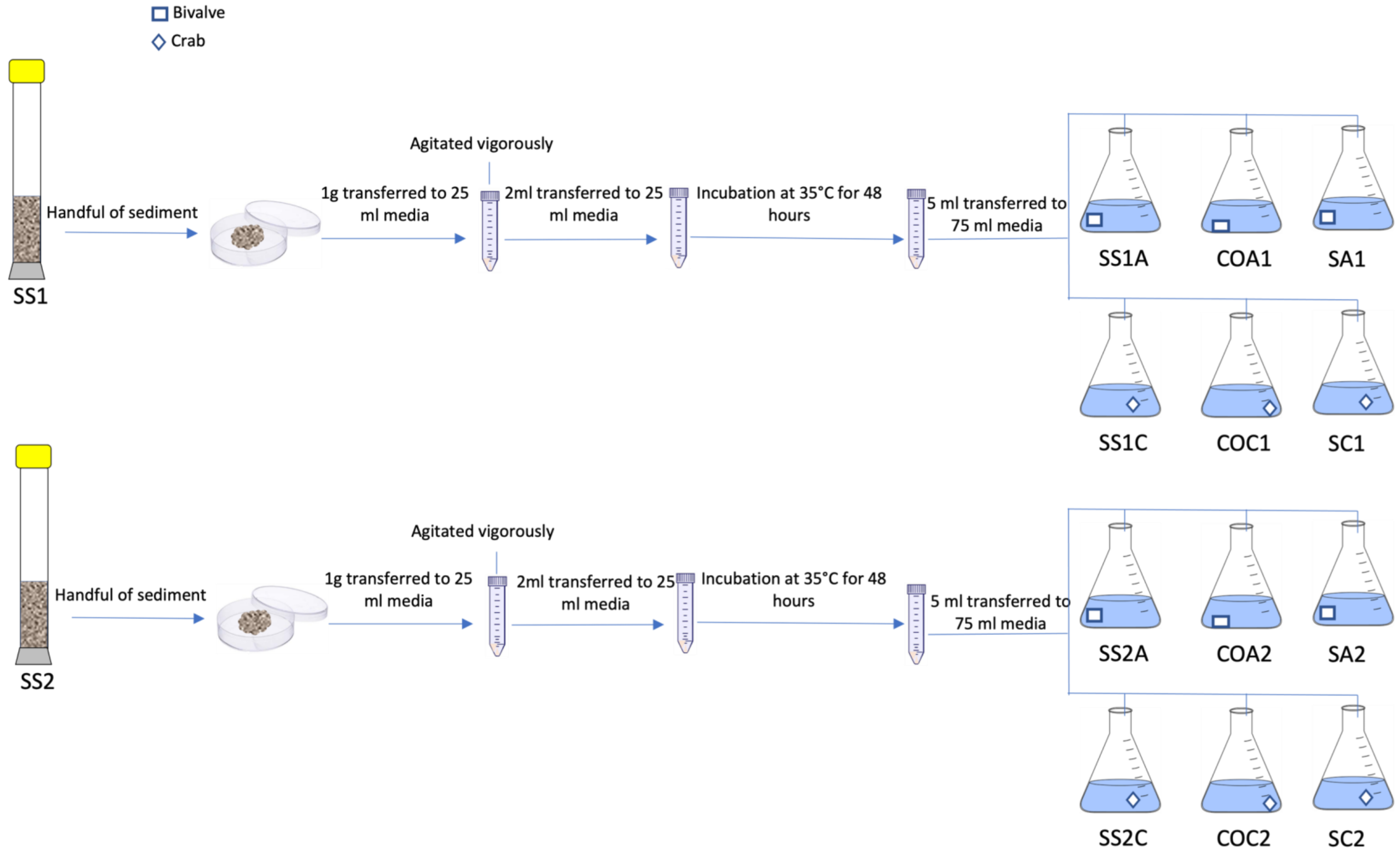

Supplementary figure 1: Schematic procedure of experiment B. The flasks SS1A, SS1C, SS2A, and SS2C are enriched from the sediments. The flasks COA1, COC1, COA2, and COC2 are the abiotic control with organic matter. The flasks SA1, SC1, SA2, and SC2 are saline abiotic control.

S5. Mineralogic, Texture, and Compositional Microstructure

S5.1 Powder X-ray Diffraction (XRD)

The mineralogy of starting materials before and after incubation was determined using X-ray diffractometer. Starting materials were pulverized into a fine powder using a pre-washed pestle and mortar that was sterilised with isopropanol. The powdered samples were loaded into the sample holder and analysed on a PHILIPS Analytical X-ray B.V. (PHILIPS, Netherlands). The detailed scanning settings were as follows: scan type Gonio with continuous mode, 2θ scanning starting from 20° to 60° with Cu-Kα radiation, and step size of 0.02° with scan speed of 0.020°/s. X'Pert Quantify software was used for scanning and HighScorePlus software was used for peak analysis and comparison with ICDD and RRUFF database.

S5.2 Scanning Electron Microscopy with Energy-Dispersive Spectroscopy (SEM-EDS)

The texture and compositional microstructures were determined using SEM-EDS. Prior to investigation, the starting materials were washed thoroughly with de-ionized water and dried for 1 hour. In experiment A (Figure 3), the samples were embedded in epoxy and polished manually while in experiment B (Figure 4) the samples were mounted on double carbon tape on an aluminium stub. All samples were coated with 10 nm of carbon coating in experiment A and gold coating in experiment B and examined using a JEOL JSM-6610LV SEM (JEOL, Japan) equipped with the X-Max EDS system (Oxford Instruments, High Wycombe, UK). INCA software (Oxford Instruments, High Wycombe, UK) was used for data acquisition and processing. The emission current was adjusted to 60 µA and voltage to 20 kV.

S6. XRD results

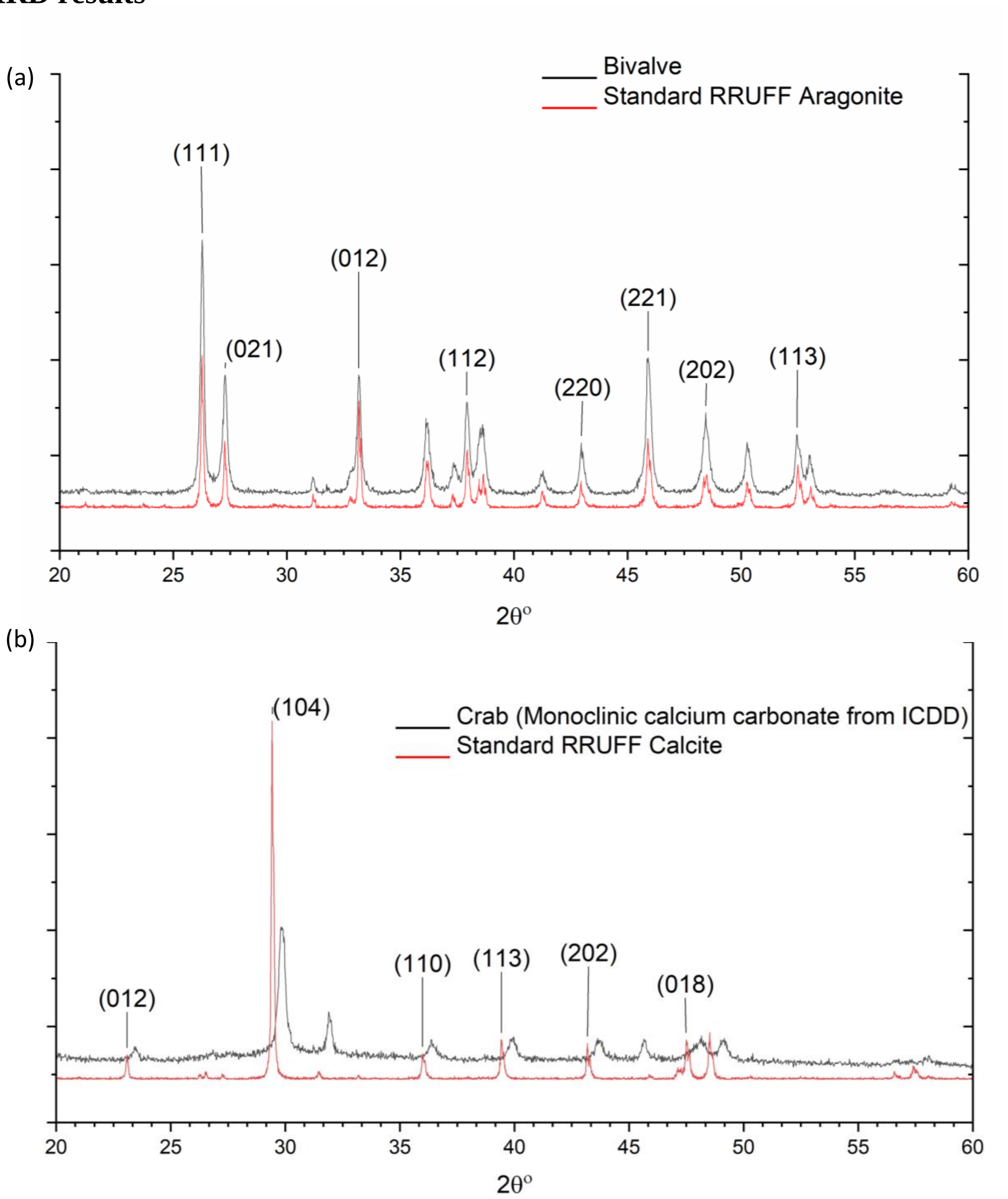

Supplementary figure 2: XRD results of starting materials after 14 days of incubation: (a) bivalve shows single-phase aragonite in comparison to RRUFF aragonite and (b) crab skeleton shows Mg-calcite with minor but varying Mg concentration in comparison to standard calcite from RRUFF database.

S7. SEM-EDS results

S7.1 Experiment A

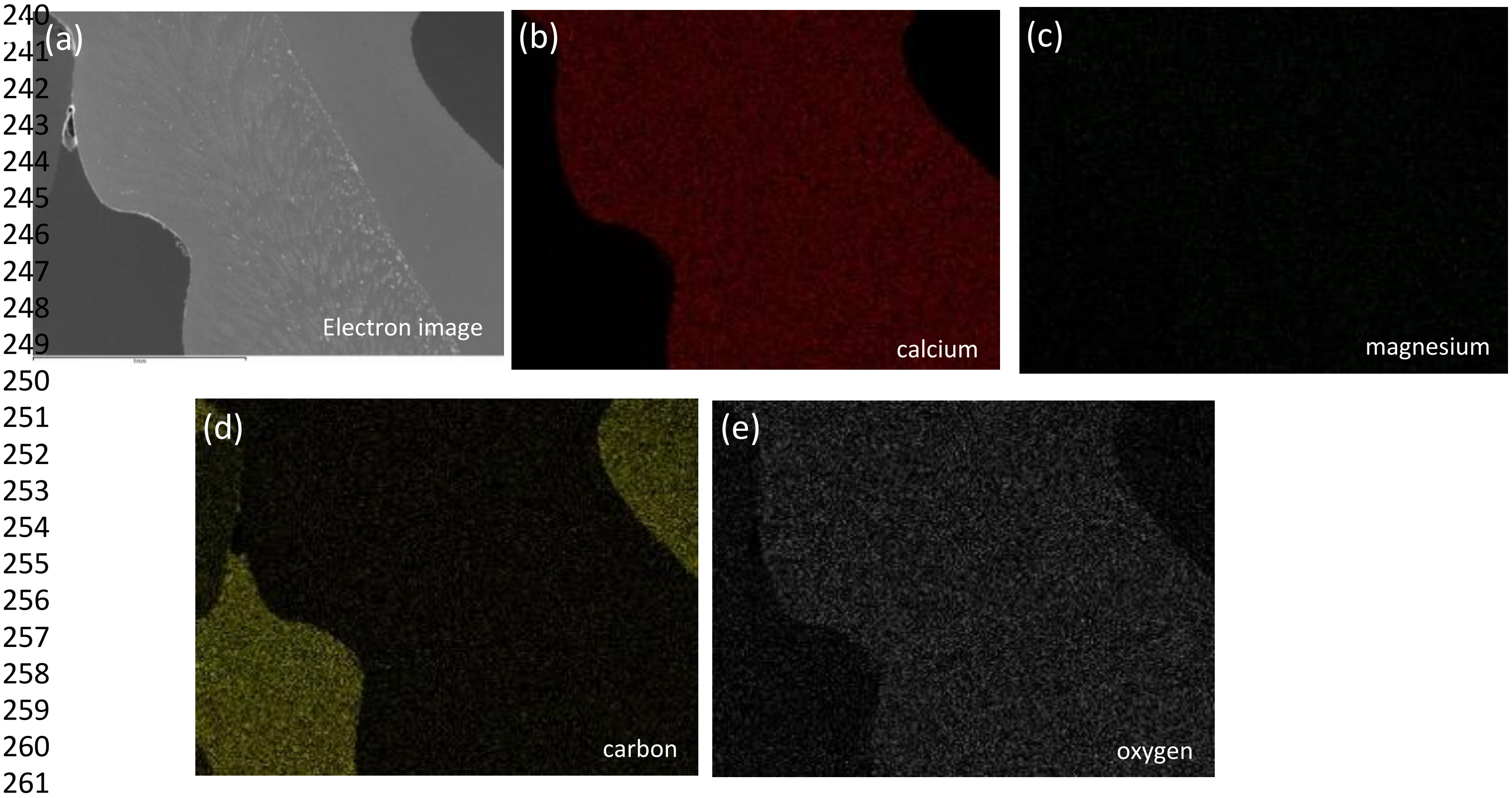

Supplementary figure 3: Textural characterization and microchemical analysis of a representative bivalve sample: (a) electron image showing minor heterogeneous color due to NaCl precipitation. Panels (b), (c), (d), and (e) are EDS mapping of calcium, magnesium, carbon, and oxygen, respectively. The figure shows no porosity or cavities after incubation neither diagenetic process related to magnesium replacing calcium in the bivalve shell.

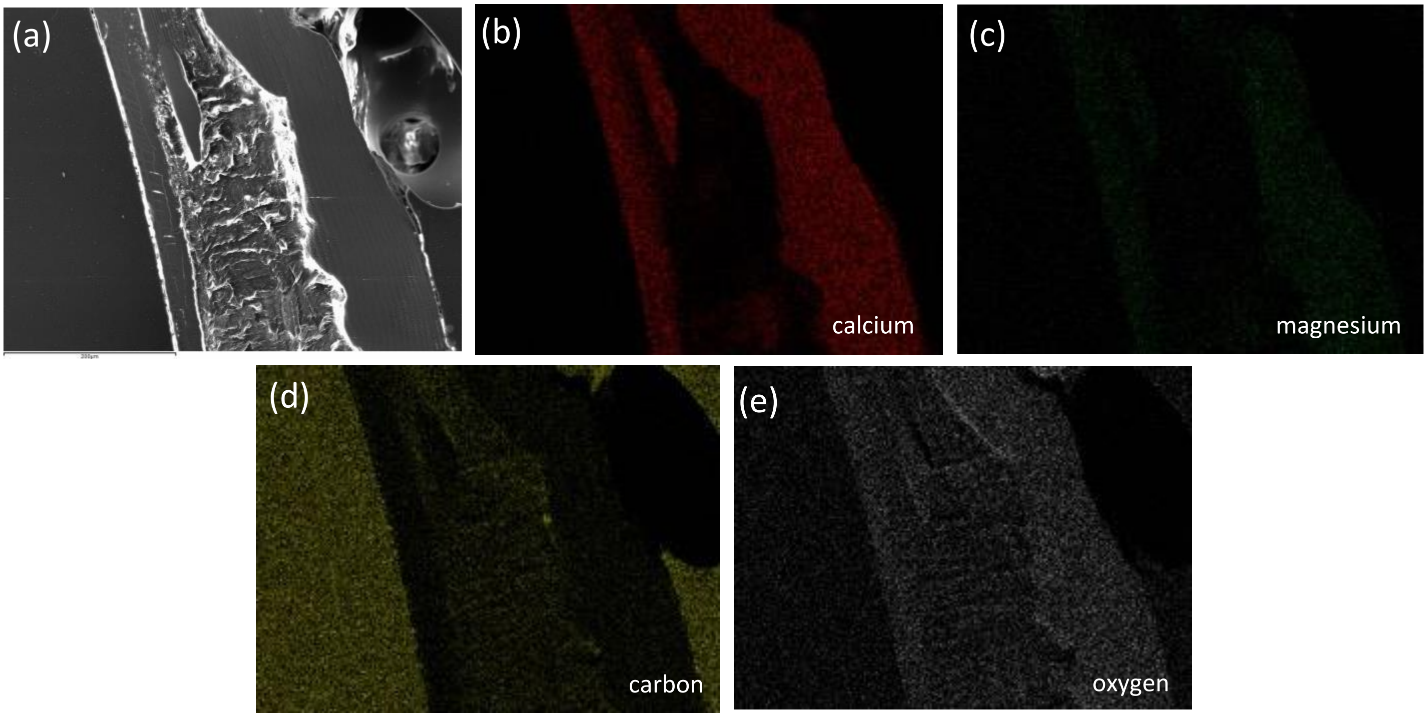

Supplementary figure 4: Textural characterization and microchemical analysis of a representative crab sample: (a) electron image showing minor heterogeneous color due to NaCl precipitation. Panels (b), (c), (d), and (e) are EDS mapping of calcium, magnesium, carbon, and oxygen, respectively. The figure shows no porosity or cavities after incubation neither diagenetic process related to magnesium replacing calcium in the crab skeleton.

S7.2 Experiment B

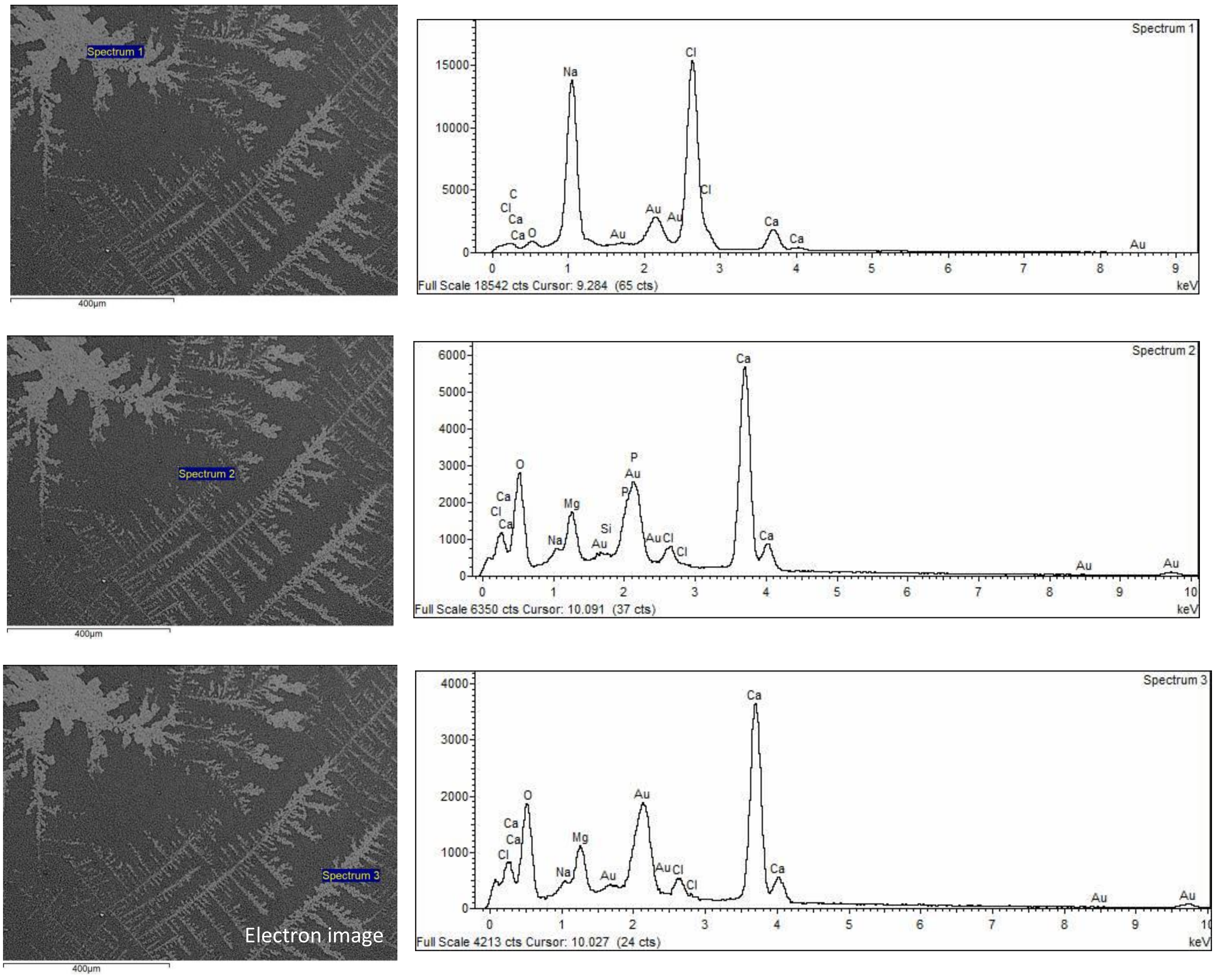

Supplementary figure 5: SSA1 shows replacement features of NaCl within bivalve shell

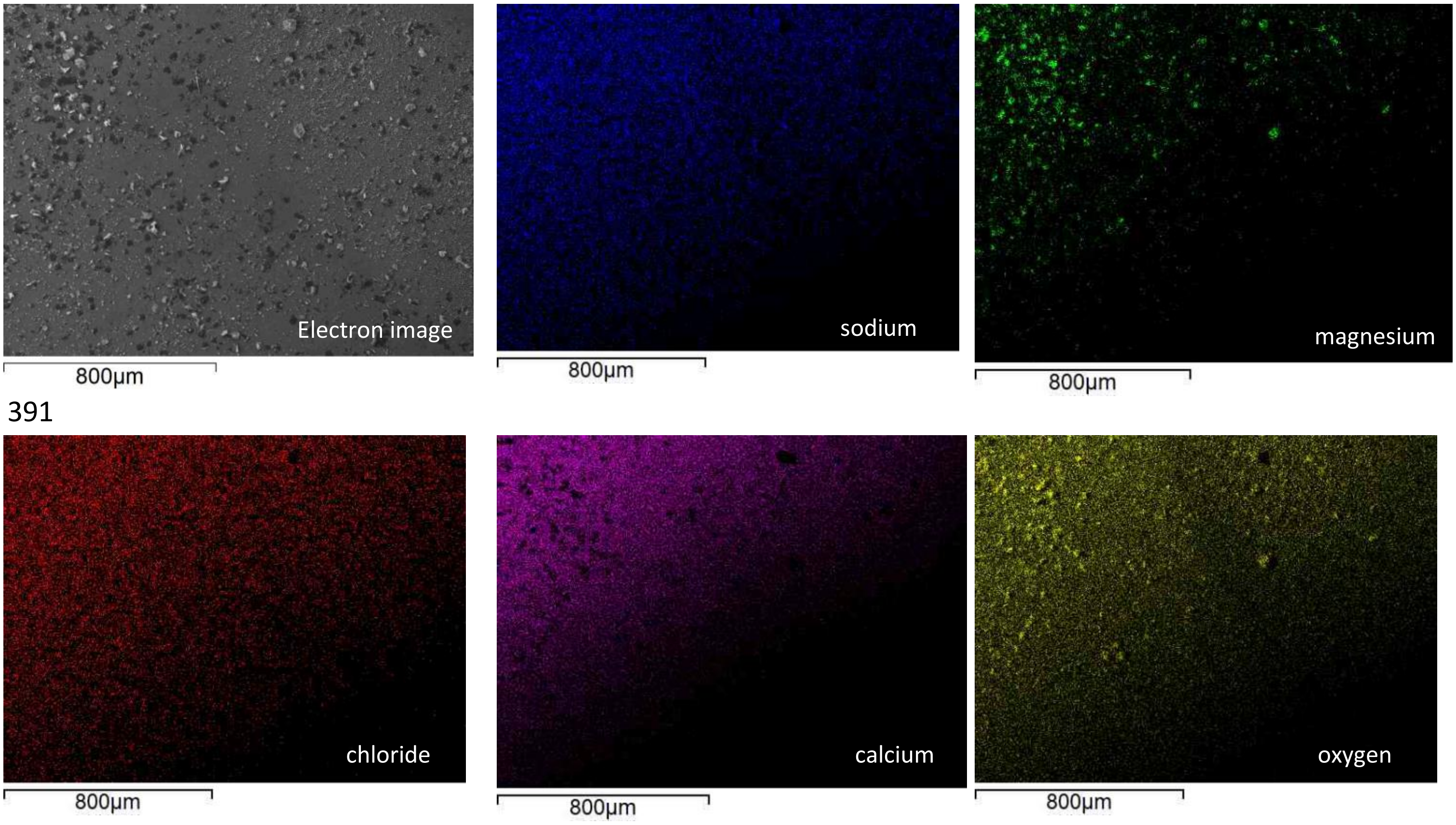

Supplementary figure 6: SS2A shows Mg precipitates but no replacement features

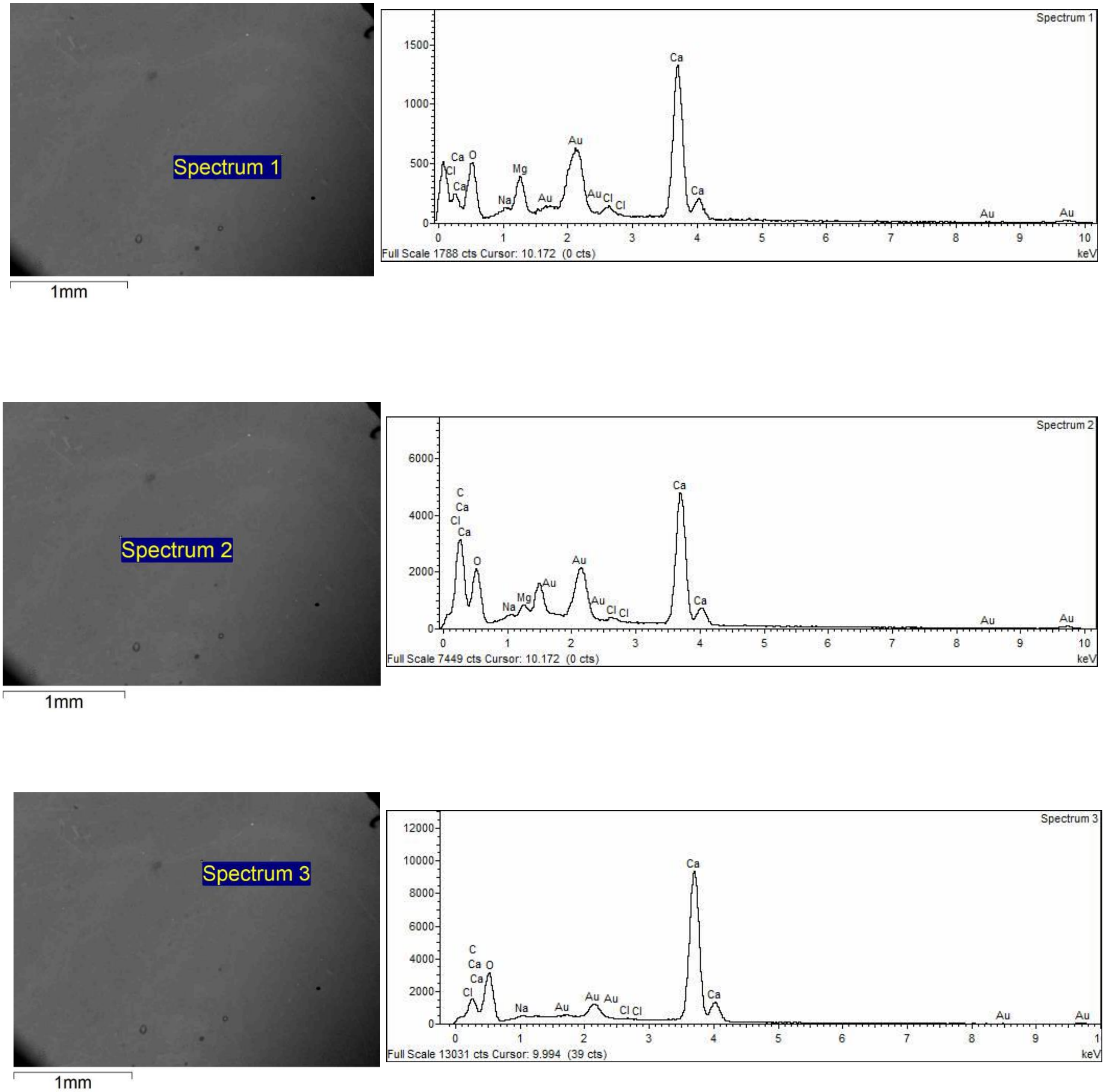

Supplementary figure 7: Absence of replacement features in COA1

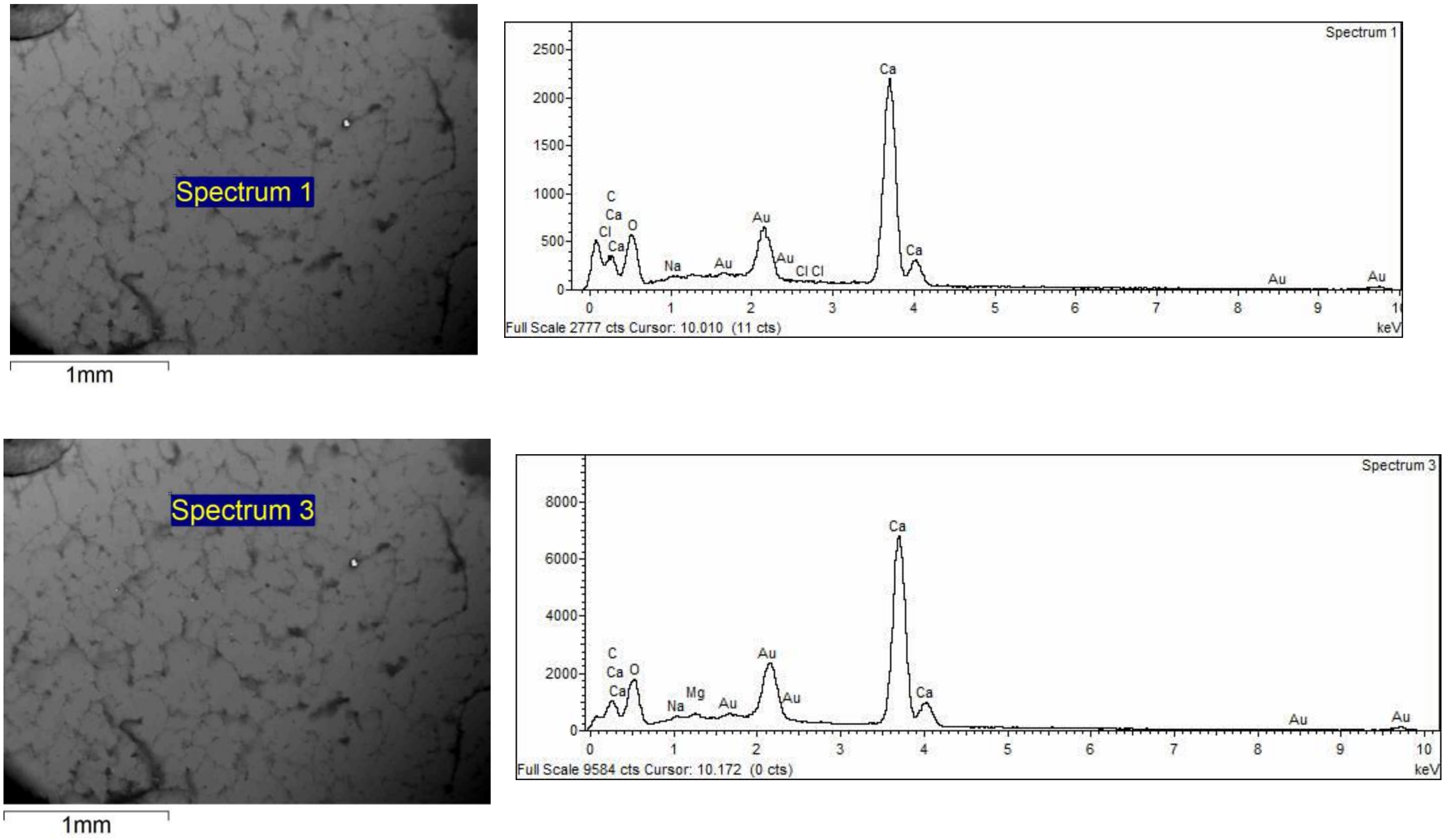

Supplementary figure 8: Heterogenous texture in COA2 irrelevant to Mg replacement

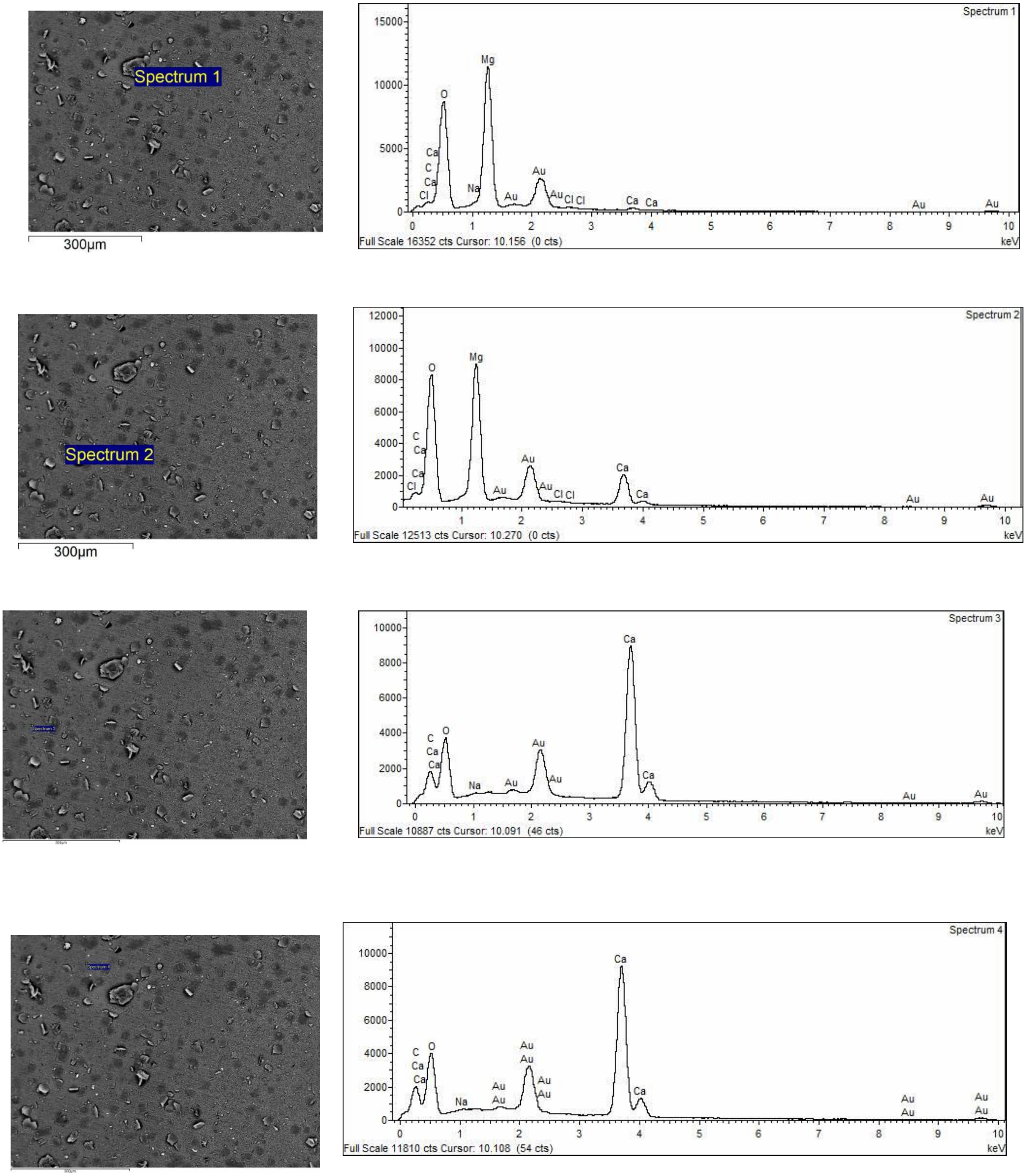

Supplementary figure 9: Mg-carbonate precipitates in SA1

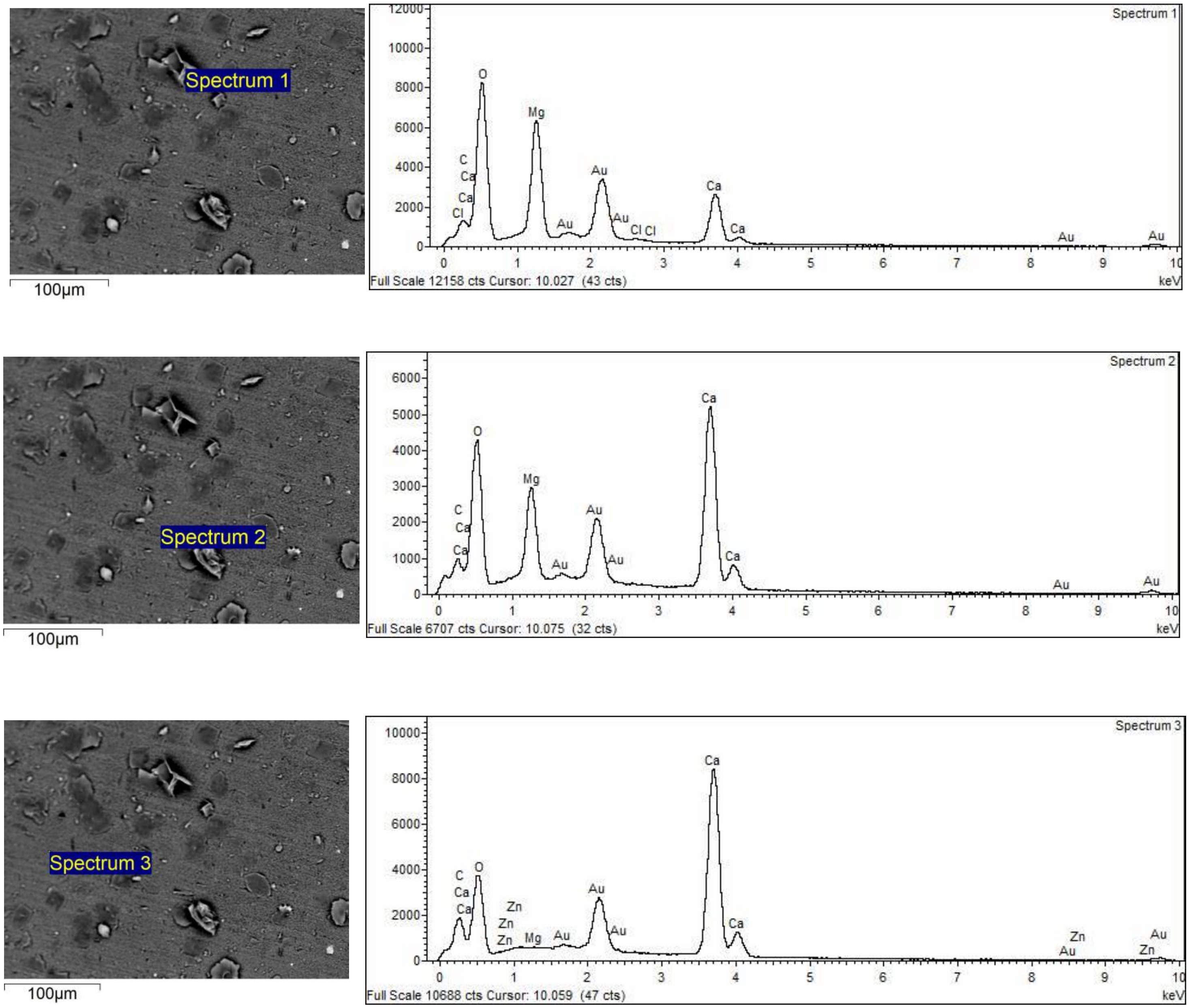

Supplementary figure 10: Mg-carbonate precipitates in SA2

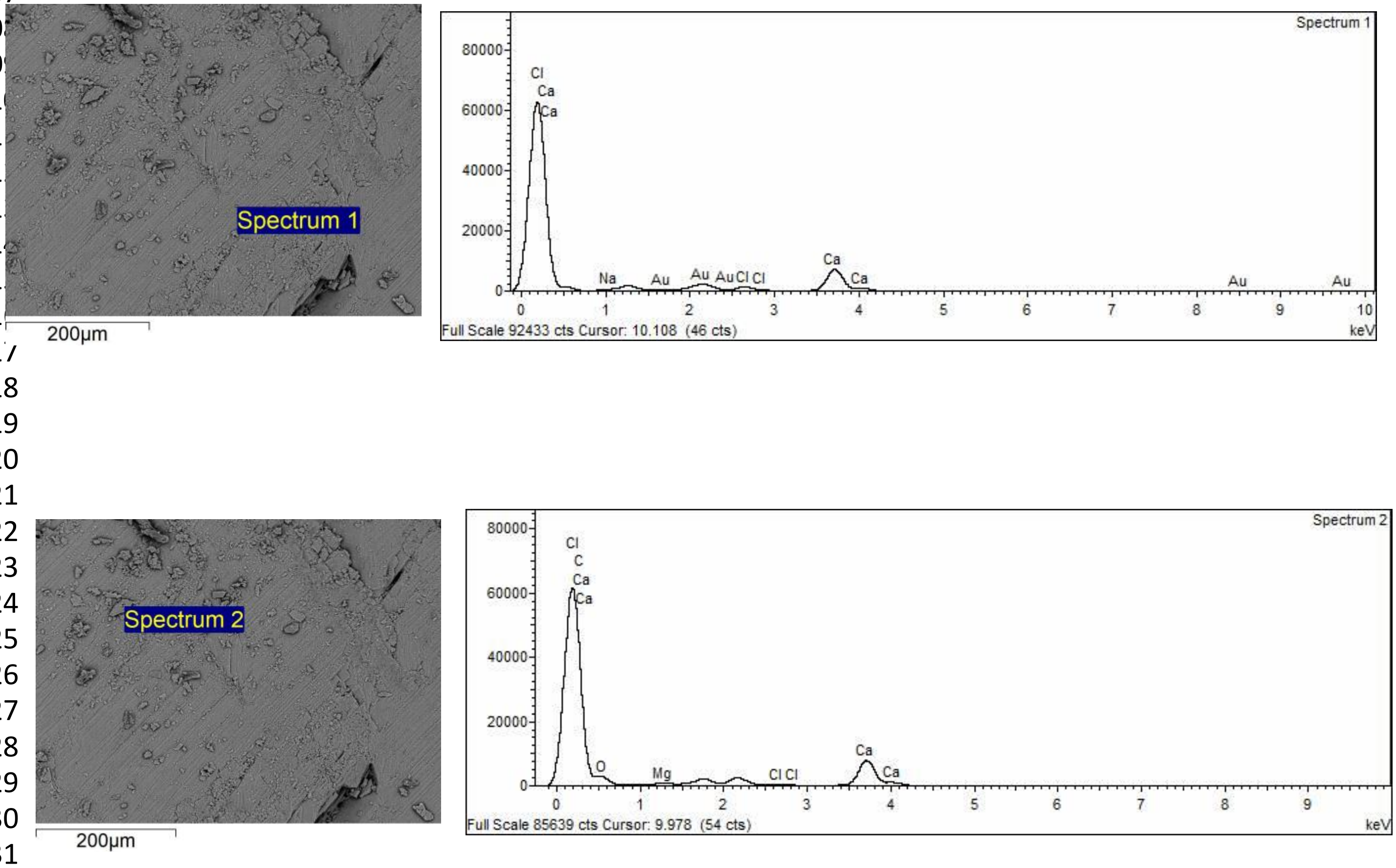

Supplementary figure 11: Absence of replacement feature in SS1C

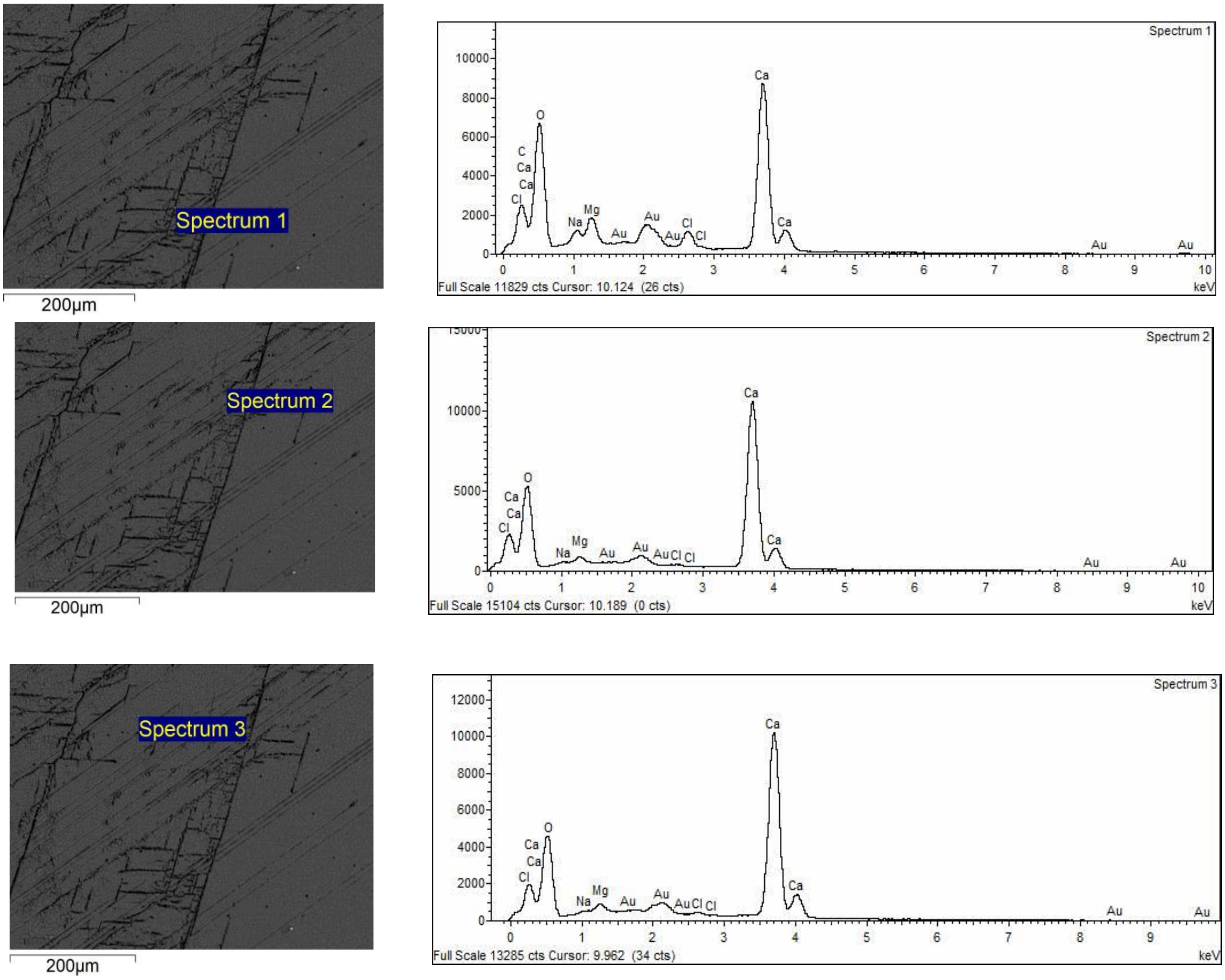

Supplementary figure 12: Heterogenous texture in SS2C irrelevant to Mg replacement

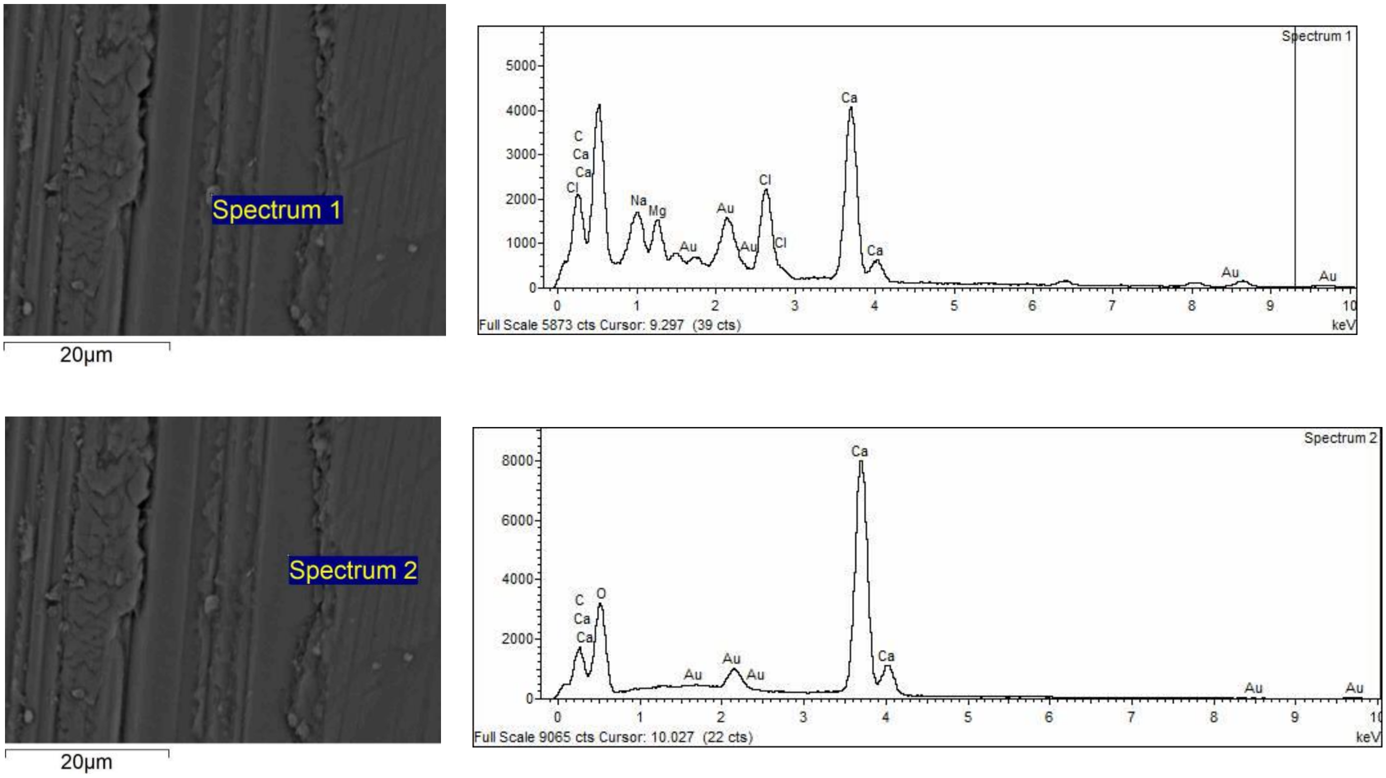

Supplementary figure 13: Precipitates of Mg-carbonates in COC1
